# Safety Aspects and Procedural Characteristics of Ambulatory Diagnostic Cerebral Catheter Angiography

**DOI:** 10.1007/s00062-023-01345-4

**Published:** 2023-09-15

**Authors:** Lars Behrens, Andreas Adam, Anna Rubeck, Stefan Schiele, Gernot Müller, Yalda Abrishami, Ansgar Berlis, Christoph J. Maurer

**Affiliations:** 1 Department of Diagnostic and Interventional Neuroradiology, University Hospital Augsburg, Stenglinstr. 2, 86156 Augsburg, Germany; 2 Department of Radiology, Hospitals Aichach and Friedberg, Friedberg, Germany; 3 Institute of Mathematics, University of Augsburg, Augsburg, Germany

**Keywords:** Cerebral arteries, Cerebral ischemia, Angiographic complications, MRI, Diffusion-weighted imaging

## Abstract

**Purpose:**

Diagnostic cerebral catheter angiography is used to assess a variety of neurovascular pathologies especially in patients before and after endovascular neurointerventional treatment. In many centers diagnostic cerebral angiographies are performed with the patient staying for one night in the hospital because there are not yet sufficient data on the safety of ambulatory cerebral angiography. At the same time hospitals face a growing demand to perform ambulatory medical procedures.

**Methods:**

A total of 426 ambulatory diagnostic cerebral angiographies were retrospectively analyzed. Technical details of the angiographies were analyzed to identify procedural risk factors.

**Results:**

Out of 426 patients 14 (3.3%) had some form of complication, 3 developed minor transient neurological symptoms, 1 patient developed Quincke’s edema probably as an adverse reaction to contrast agent, 1 patient had an asymptomatic carotid dissection and 1 had a fall of unknown etiology. Of the 14 complications 8 were puncture site complications with 1 re-bleeding, 1 dissection, and 6 minor complications, 421 punctures were femoral, 3 radial and 2 brachial. Out of 333 patients with magnetic resonance imaging (MRI) after angiography 21 showed focal diffusion-weighted imaging (DWI) lesions but none of these lesions were symptomatic. The rate of DWI lesions was significantly higher in selectively angiography territories than in other territories. The use of a Simmons 2 catheter significantly increased the rate of DWI lesions (*p* = 0.047), whereas 3D rotational angiography did not (*p* = 0.55). The rate of DWI lesions per selectively accessed vessel was 4.6% with a higher rate in the anterior than in the posterior circulation.

**Conclusion:**

Diagnostic cerebral catheter angiography can be safely performed in an ambulatory setting.

## Background and Purpose

Diagnostic cerebral catheter angiography is a valuable tool in assessing a variety of neurovascular pathologies. Although noninvasive angiography techniques such as computed tomography angiography and magnetic resonance angiography have replaced conventional cerebral angiography in many cases, conventional diagnostic angiography is still the method of choice for a variety of indications especially in patients before and after endovascular treatment for aneurysms, intracranial stenosis, arteriovenous malformations or dural arteriovenous fistulas.

Many diagnostic cerebral angiographies are still performed with the patient staying in the hospital for at least one night after angiography for safety reasons. Main concerns are complications at the puncture site and cerebral ischemic lesions [1, 2]. Serious complications at the puncture site may include bleeding, pseudoaneurysms, arteriovenous fistulas, or arterial dissections. At the same time hospitals face a growing demand to perform medical procedures in an ambulatory setting.

Despite the possible complications of catheter angiography, coronary angiography and even some percutaneous coronary interventions have been performed in an ambulatory setting in the past years with proven feasibility and safety [3, 4].

There is a general trend in all parts of medicine to shift diagnostic or interventional procedures from an inpatient to an outpatient basis. This is usually more cost effective but it must be assured that no additional risks arise when compared to inhospital procedures. Therefore, the main objective of this study was to confirm that outpatient diagnostic cerebral angiography is safe.

## Material and Methods

We retrospectively reviewed images and medical charts of patients who received an ambulatory diagnostic cerebral angiography at our institution from 2016 to 2021. In the ambulatory setting patients received angiography in the morning and stayed in an outpatient ward for 6h after the procedure. In all angiographies catheters were continuously flushed with heparinized saline solution. Before discharge each patient was revisited by the neuroradiologist and was examined for newly developed neurological symptoms and complications at the puncture site or otherwise. All patients had a clinical follow-up again in our outpatient clinic within days to weeks after diagnostic angiography. Neurological condition and possible puncture site complications were documented.

Arterial puncture was performed at the common femoral artery in 421 patients, at the radial artery in 3 patients, and at the brachial artery in 2 patients.

In 372 of the 421 patients (88.4%) with groin puncture a 5F introducer sheath was used, in 49 patients (11.6%) a 6F introducer sheath. In two of the three patients with radial puncture a 5F introducer sheath was used, in one patient a 4 F introducer sheath. In the two patients with brachial puncture a 5F introducer sheath was used.

After completion of angiography 307 patients received an Exoseal vascular closure device (Cordis, Bridgewater, NJ, USA) with subsequent manual compression and application of a compression bandage, 65 patients received an Angioseal vascular closure device (Terumo Corporation, Tokio, Japan) with or without compression bandage, and in 49 patients the puncture site was manually compressed only with subsequent application of a compression bandage. The three patients with radial artery puncture received manual compression with a QuikClot Radial hemostatic gauze (Teleflex, Wayne, PA, USA) and subsequent mild pressure bandage.

All 426 angiographies were retrospectively analyzed with special emphasis on patient safety. Technical details of the angiographies were also analyzed to identify procedural risk factors.

Diagnostic angiographies were performed with a biplane system (Axiom Artis, Siemens Healthineers, Erlangen, Germany).

Out of 426 patients 326 had follow-up MRI with diffusion-weighted imaging (DWI) within 21 days after angiography (median 9.0 days). Another 7 patients had an MRI with contrast-enhanced imaging within an extended time frame of 22–42 days. Thus, 333 of 426 patients had an MRI capable of detecting or excluding cerebral ischemia after angiography.

The MRI examinations were performed using 1.5 T scanners (Magnetom Avanto or Magnetom Aera, Siemens Healthineers).

In this study we also checked for possible procedural risk factors. Examination protocols and reports as well as archived angiographic images were analyzed. Archived MRI images were analyzed for ischemic lesions on DWI. For evaluation of risk factors for DWI lesions we recorded the following parameters: patient age, patient sex, duration of procedure, years of angiographic experience of the neuroradiologist, use of a catheter with Simmons 2 configuration. We also examined whether DWI lesions occurred in a vascular territory that was angiographied or in non-angiographied territories. For this purpose anatomic variants such as embryologic origin of the posterior cerebral artery were taken into account.

Patient characteristics were summarized by descriptive statistics. For comparison between patients with DWI lesions after angiography and those without we used a Wilcoxon rank-sum test for continuous variables and for categorial variables a Fisher exact test or a χ^2^-test was applied. To determine the effect size, Cohen’s W was calculated. All statistical analysis was performed using R 4.1.2 (R Foundation for Statistical Computing, Vienna, Austria) and a *p* < 0.05 was considered statistically significant.

## Results

### Patients

A total of 426 patients received an ambulatory diagnostic cerebral angiography at our institution from 2016 to 2021. Of the patients 280 were female (65.7%), 146 male (34.3%) and 402 patients underwent routine follow-up angiography after endovascular treatment of intracranial pathologies according to the standards of our department (348 had aneurysms, 25 dural arteriovenous fistulas, 20 arteriovenous malformations, 4 carotid cavernous fistulas and 5 intracranial stenosis). Of the patients 24 had a diagnostic angiography without prior treatment to exclude or confirm vascular pathologies suspected on MRI.

Out of 426 patients 14 (3.3%) had some kind of complication, 8 of the 14 complications were puncture site complications with 1 re-bleeding, 1 dissection, and 6 had minor puncture site complications, 421 punctures were femoral, 3 radial and 2 brachial. Three other patients developed minor transient neurological symptoms. One patient developed Quincke’s edema probably as an adverse reaction to contrast agent, one patient had an asymptomatic carotid dissection and one had a fall of unknown etiology.

### Complications Related to Vessel Puncture

Three patients with puncture of the common femoral artery presented to the emergency department after discharge with groin pain. In all three cases there was no pathological finding on physical examination and ultrasound besides minor hematoma.

One patient had a dissection of the common femoral artery related to vessel puncture. The patient was without symptoms at discharge but was readmitted to the hospital 24 days after angiography due to intermittent claudication of the right lower leg for several days. An occlusion of the popliteal artery with pre-existing stenosis was diagnosed and the patient was treated the same day endovascularly with angioplasty and stenting. The dissection was found to be without hemodynamic compromise and was therefore not specifically treated. Claudication did not reappear after stenting.

One patient with puncture of the common femoral artery and Angioseal closure device had persistent dysesthesia in the groin. One patient with puncture of the radial artery had temporary dysesthesia in the thumb.

In one patient the Exoseal closure device appeared at the skin surface several days after angiography without any complication at the puncture site.

One patient suffered from rebleeding in the groin immediately after removal of the compression bandage 6 h after application of an Angioseal 6F vascular closure device and subsequent placement of the compression bandage. This patient was under single antiplatelet therapy with acetylsalicylic acid. Manual compression was applied immediately followed by replacement of a compression bandage for 12 h. Afterwards there was no more bleeding. A pseudoaneurysm or arteriovenous shunt was ruled out by ultrasound. The patient had to stay one night unscheduled in the hospital and could be discharged 1 day after angiography.

### Other Complications

One patient developed Quincke’s edema probably as an adverse reaction to contrast agent after discharge and was readmitted to hospital. Treatment was successful without sequelae.

One patient was admitted to the emergency department the day after angiography because of a fall of unknown etiology. The patient had no neurological deficits.

One patient had a dissection of the internal carotid artery after diagnostic series were performed including 3D rotational angiography. The dissection was without hemodynamic compromise and was treated with acetylsalicylic acid 100 mg once daily orally for 6 months. The patient was completely asymptomatic at all times.

### Neurological Deficits

All 426 patients had clinical follow-up after diagnostic angiography because they were examined before discharge from the outpatient department and seen again later in our outpatient clinic. Of the 426 patients 3 (0.7%) suffered from transient minor neurological deficits (in 2 patients memory impairment and word finding disorder lasting for 2 days or for a few hours, in 1 patient dysesthesias lasting for 1 day). None of these 3 symptomatic patients had DWI lesions and none of the 21 patients with DWI lesions on MRI had neurological symptoms.

### DWI Lesions

**Fig. 1:**
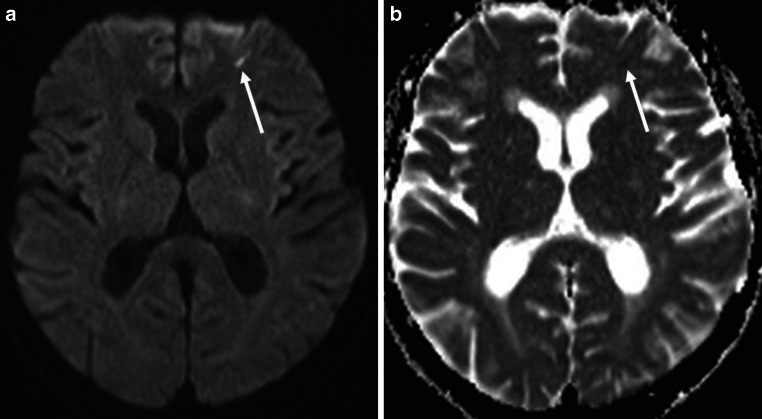
Axial diffusion-weighted imaging (DWI) of the brain with a small DWI lesion (*arrows*) in the left frontal lobe 4 days after angiography. **a** DWI b-value 1000, **b** Apparent Diffusion Coefficient (ADC)

Out of 426 patients 333 (78.2%) had an MRI after angiography, 21 of these 333 (6.3%) patients had DWI lesions on MRI most likely attributable to diagnostic angiography. None of these lesions were symptomatic. The DWI lesions were typically small (Fig. 1) and had a mean maximum diameter of 3.7 mm with a standard deviation of 1.7 mm (range 1.8–8.5 mm).

Of the 21 patients with DWI lesions 17 had DWI lesions in only 1 vascular territory, 4 had DWI lesions in 2 vascular territories, making this a total of 25 vascular territories affected. In 13 cases the territory of the left internal carotid artery was affected, in 8 cases the territory of the right carotid artery and in 4 cases the vertebrobasilar territory (Table 1). In three of the latter four cases DWI lesions occurred in the basilar artery territory making a distinction between left or right vertebral artery not possible, the other one of these four cases occurred in the territory of the left vertebral artery.

**Table 1:** Anatomic distribution of all diffusion-weighted imaging (DWI) lesions

Total number of territories with DWI lesions	25
Territory of left carotid artery	13 (52%)
Territory of right carotid artery	8 (32%)
Territory of vertebral arteries	4 (16%)

#### Patient Age and Sex

Mean patient age in the group of 333 patients with follow-up MRI was 52 years with a range from 18 to 76 years. Mean age of the 21 patients with DWI lesions was 58 years with a range from 43 to 71 years. Mean age of the 312 patients without DWI lesions was 52 years with a range from 18 to 76 years. Patients older than 52 years had a significantly higher rate of DWI lesions than those younger than 52 years (9.3% vs. 2.6%, *p* = 0.001, effect size = 0.322).

The rate of DWI lesions in female patients was 6.1% (14/229) and in male patients 6.7% (7/104), showing no statistically significant difference (*p* = 0.83).

#### Do DWI Lesions Occur More Often in Vessels with Selective Angiography?

We explored whether DWI lesions occurred in vascular territories with selective angiography or in other territories. The 333 patients with MRI follow-up had a total of 549 territories (left or right carotid artery, left or right vertebral artery) selectively angiographied. Of the 25 territories with DWI lesions 19 (76.0%) were territories selectively angiographied and 6 territories (24.0%) were territories not selectively angiographied. The difference in the rate of DWI lesions in selectively angiographied territories versus territories not angiographied was statistically significant (*p* = 0.014).

The rate of DWI lesions per vessel accessed was 25/549 (4.6%) in total or 19/549 (3.5%) when only DWI lesions in accessed vessels were counted (Table 2).

**Table 2:** Overview of accessed vessels in the study cohort and rate of diffusion-weighted imaging (DWI) lesions in the respective territories

Vessel	Number of vessels accessed	Number of territories with DWI lesions	Rate of DWI lesions (%)
Left carotid	212	11	5.2
Right carotid	207	6	2.9
Left and right vertebral	130	2	1.5
Total	549	19	3.5

The rate of DWI lesions in accessed vessels were in the left carotid artery 5.2% (11/212), in the right carotid artery 2.9% (6/207), and in the left and right vertebral artery combined 1.5% (2/130). These differences were not statistically significant.

#### Does the Use of a Simmons 2 Catheter Influence the Rate of DWI Lesions?

In 45 of 333 patients (13.5%) with follow-up MRI a Simmons 2 catheter had to be used because of an unfavorable supra-aortic vascular anatomy. Of the 45 patients with Simmons 2 catheter 6 had a DWI lesion (13.3%) and 15 of 288 without Simmons 2 catheter had a DWI lesion (5.2%) (Table 3). The use of a Simmons 2 catheter had an increased rate of DWI lesions compared to angiographies without the use of a Simmons 2 catheter (*p* = 0.047, effect size = 0.114).

**Table 3:** The rate of diffusion -weighted imaging (DWI) lesions was significantly higher when a Simmons 2 catheter was used (*p* = 0.047)

	Number	Percent
*Patients with magnetic resonance imaging follow-up*	333	–
*Total with Simmons 2 catheter*	45	–
Simmons 2 with DWI lesions	6	(6/45) 13.3%
*Total without Simmons 2 catheter*	288	–
With DWI lesions	15	(15/288) 5.2%

All six Simmons 2 patients with DWI lesions were asymptomatic. four of the six patients had DWI lesions in only one vascular territory, two had DWI lesions in two vascular territories, making this a total of eight vascular territories affected. Of these eight territories four were territories entered with the Simmons 2 catheter (50.0%) and four territories were unrelated to direct entry of the catheter (50.0%) with one of the latter four being the territory of the left vertebral artery in which the catheter was introduced for configuration.

#### Does the Rate of DWI Lesions with Simmons 2 Catheter Direct Access Vary with the Vessel Entered?

The left carotid artery was entered with the Simmons 2 catheter in 35 cases, in 3 of these a DWI lesion in this territory was detected (8.6%). The respective values were for the right carotid artery 1/18 (5.6%), for the left vertebral artery 0/1 (0.0%), and for the right vertebral artery 0/4 (0.0%).

#### Does 3D Rotational Angiography Influence the Rate of DWI Lesions?

A 3D rotational angiography was performed in 323 of 549 vessels selectively angiographied (58.8%) and 16 of the 25 territories with DWI lesions had 3D rotational angiography (64.0%). The rate of DWI lesions in vessels with 3D rotational angiography was 16/323 (5.0%) and 9/226 (4.0%) without 3D rotational angiography. This difference was not statistically significant (*p* = 0.41).

Of the four DWI lesions in the posterior circulation none were in a territory examined with 3D rotational angiography and 50% (2/4) had a vertebral artery directly accessed.

#### Angiographer’s Level of Experience

Mean years of angiographic experience of the neuroradiologist in the group of 333 patients with follow-up MRI was 12.3 years with a range from 0 to 34 years with 0 being within the first year of experience. Of the angiographies 63/333 (18.9%) were performed by neuroradiologists with less than 2 years of angiographic experience and 219/333 (65.8%) by neuroradiologists with more than 5 years of angiographic experience. Mean years of experience of the neuroradiologist of the 21 patients with DWI lesions was 11.3 years with a range from 0 to 32 years. Mean years of experience of the neuroradiologist of the 312 patients without DWI lesions was 12.4 years with a range from 0 to 34 years.

There was a statistically significant correlation between the years of experience of the angiographer and the duration of the angiography with shorter duration when the angiographer was more experienced (*p* < 0.001) but the angiographer’s level of experience did not significantly differ between patients with DWI lesions and without (*p* = 0.67).

## Discussion

This study demonstrates the safety of ambulatory diagnostic cerebral catheter angiography. This is an important finding as there is growing demand for ambulatory medical care. In 426 consecutive patients there were no permanent neurological symptoms attributable to angiography and only a few minor transient neurological symptoms. Puncture site complications occurred at a low rate despite the fact that about two thirds of patients received antiplatelet or anticoagulatory medication. with most of them being groin pain without pathological findings. There was no complication that would not have occurred in hospital or that could have been managed better within a primary inhospital setting.

Only 1 of 421 patients with puncture of the common femoral artery had rebleeding. This bleeding rate is low compared with the literature where bleeding rates of up to 6.4% after use of vascular closure devices and up to 6.8% after manual compression are reported [5]. It is likely that bleeding rates were lower in our series because all patients regardless of the use of a closure device were required to keep bedrest for 6h. In addition, many patients in other studies which showed higher bleeding rates were patients who underwent interventions rather than diagnostic angiography and therefore many of these patients received aggressive antiplatelet therapy and larger introducer sheaths. There was no case of retroperitoneal hematoma in our series. The low rate of bleeding complications in our series was despite many patients being on antiplatelet therapy thus demonstrating the safety of the use of vascular closure devices in an ambulatory setting.

Three patients presented to the emergency department after discharge because of groin pain. None of these patients had a complication that needed treatment. Temporary groin pain is a common finding after femoral artery puncture and the level of pain which leads patients to visit the emergency department is very variable.

With respect to the one patient with dissection of the right common femoral artery it is unclear whether the popliteal occlusion was a thromboembolic event caused by the dissection. As there was a pre-existing stenosis it might have also been unrelated to the dissection. Fortunately, the patient could be treated with angioplasty and stenting and had no sequelae; however, this event demonstrates the need to instruct patients before discharge to report to the hospital immediately in any such event as such complications are potential risks in ambulatory angiography.

None of the patients developed a femoral artery pseudoaneurysm or a femoral arteriovenous fistula, whereas the literature reports a rate of femoral artery pseudoaneurysms of 0.1–0.2% after puncture for diagnostic angiography [6].

We cannot provide sufficient data on whether radial artery access results in more or fewer puncture site complications as almost all of our diagnostic angiographies were performed with femoral artery access. Radial artery access has only recently become a more widely used alternative for cerebral angiography and intervention [7]. Radial artery access could potentially lead to less hematomas and more patient comfort, maybe also to faster discharge within the ambulatory setting. Patients can be mobilized immediately after angiography. Radial access has been shown to be noninferior to femoral access in diagnostic cerebral angiography [8]. Disadvantages of radial access include potential vasospasms of the radial artery and more difficulties if access to all four cerebral vessels is required. This access route, however, might become in eligible patients the preferred choice in cerebral angiography when an ambulatory setting is desired.

We found no evidence of ambulatory diagnostic cerebral angiography leading to higher rates of puncture site complications.

Dissection of cervical arteries during diagnostic cerebral angiography are reported to occur in 0.39% of cases, most of them in the vertebral arteries and the vast majority remain asymptomatic [9]. We only had 1 dissection in 426 patients (0.23%) which occurred in an internal carotid artery and remained asymptomatic.

Our rate of DWI lesions (6.3%) appears relatively low compared to the literature where rates of 25.8% are reported for the group of patients with diagnostic cerebral angiography [10]. The incidence in that study might have been higher than ours because in-house patients for example with vasculitis were included who are probably more prone to ischemia than otherwise healthy patients, whereas for the small group of five patients with control angiography after aneurysm coiling no DWI lesions were reported. Anyhow, a more recent meta-analysis again showed a similarly high incidence of DWI lesions after diagnostic cerebral angiography of 24.7% [11].

Nonetheless, our relatively low rate of DWI lesions might also be attributable to the continuous flushing of the catheter with heparinized saline solution which should reduce the likelihood of embolic events. In addition, many of our patients had antiplatelet monotherapy with acetylsalicylic acid 100 mg once daily or even dual antiplatelet therapy due to the nature of the initial neurointervention (e.g., stent-assisted aneurysm treatment or treatment with flow diverters), a medication that should also reduce thromboembolic events.

It has to be taken into account that the likelihood of DWI lesions increases with the number of arteries accessed. While certain indications for diagnostic cerebral angiography usually lead to complete cerebral angiography with both internal carotid arteries and both vertebral arteries accessed, most of our patients received only limited angiography with one or two arteries being accessed because in the majority of cases the reason for angiography was follow-up after aneurysm treatment. We therefore calculated the risk of the appearance of DWI lesions per vessel accessed to be 25/549 (4.6%). Extrapolation of this one-vessel risk to a four-vessel angiography leads to a theoretical rate of DWI lesions of 17.2% thus being somewhat lower than but consistent with the abovementioned rate of 24.7% in the literature.

As MRI was performed after a median time of 9 days after angiography it is likely that in some cases DWI lesions had already disappeared on imaging. Therefore, the rate of DWI lesions is probably somewhat underestimated.

Fortunately, all of our DWI lesions were asymptomatic but the possible occurrence of these lesions should be kept in mind when performing cerebral angiography. The functional architecture of the brain theoretically allows for even small lesions to potentially lead to severe and lasting functional damage.

Angiography-associated cerebral DWI lesions can be due to embolization of dislodged plaque or iatrogenic air embolism, due to local thrombosis at the catheter tip with thromboembolization, or due to catheter-induced vasospasm.

Patient age proved to be a significant risk factor for the development of DWI lesions. This is not surprising given the fact that older patients generally tend to have a more tortuous supra-aortic vascular architecture and more atherosclerotic lesions. The risk for embolic complications should therefore be higher in older patients and higher rates of ischemic complications after diagnostic cerebral angiography in older patients have already been reported [1].

The higher rate of DWI lesions in vascular territories that were directly accessed with the catheter is not surprising as mainly direct vessel access brings along the possibility of plaque dislocation. Possible thrombotic events at the catheter tip also primarily affect the vessel accessed; however, 24% of DWI lesions occurred in vessels that were not accessed with the catheter. The most obvious explanation for this phenomenon is the aortic arch. From here plaques or thrombi can be dislodged into any arising vessel whether or not it is directly entered. This is supported by data from cardiac interventions in which supra-aortic vessels were not accessed at all. The RETHREVA registry for example showed DWI lesions in 39% of patients after left atrial catheter ablation for atrial fibrillation with of course atrial fibrillation itself potentially contributing to the amount of DWI lesions [12].

The use of a Simmons 2 catheter proved to be a risk factor for DWI lesions. On the one hand this could be due to the catheter itself with respect to its stress on the wall of the aortic arch or configuration maneuvers in the aortic arch or in other vessels such as the left subclavian artery. On the other hand, this might be caused by the challenging vascular anatomy of these patients that made the use of the Simmons 2 catheter necessary. Gaining access into a tortuous anatomy is not only an angiographic challenge but it is also more likely to find atherosclerotic lesions in this subgroup of patients as tortuous vascular anatomy and atherosclerosis share arterial hypertension as a common risk factor.

The significantly higher risk of DWI lesions in territories selectively angiographied emphasizes the need to carefully select the vessels that have to be visualized in an individual patient. This holds especially true if a Simmons 2 catheter is needed and in older patients.

As older patients had a higher risk of DWI lesions patient age should also influence the decision on whether to perform outpatient angiography; however, as we did not observe any permanent neurological deficits after angiography age alone should not exclude patients from ambulatory angiography.

It is not surprising that performing 3D rotational angiography does not lead to a significantly higher rate of DWI lesions. The only difference to regular angiography is the larger amount of contrast agent needed and potentially slightly more pressure applied during contrast agent injection. This could possibly lead to vascular damage such as dissection. Indeed, our only case of dissection of a cervical artery occurred after 3D rotational angiography but it was only one case and therefore not statistically significant.

It was somewhat surprising that the experience level of the angiographer showed no statistically significant correlation with the rate of DWI lesions as the opposite has been reported in the literature [13]. This finding can be explained though when considering two factors. First, this was a retrospective study and there is probably a certain selection bias with angiography in patients with known difficult vascular anatomy being preferentially performed by more experienced angiographers. Second, the majority of angiographies were not performed by beginners with only 18.9% of angiographies being performed by neuroradiologists with less than 2 years of angiographic experience. It is reasonable to assume that after a few years of experience the learning curve has flattened enough to show no significant differences in noninterventional diagnostic angiography.

The high rate of female patients (65.7%) is consistent with the higher prevalence of intracranial aneurysms in women [14] as the majority of our patients had angiography for routine control after endovascular treatment for intracranial aneurysms. For example, 75.6% of the 4060 patients enrolled in the ISUIA aneurysm study were women [15]; however, we did not find any sex-related difference in complication rates.

A limitation of this study is its retrospective character. On the other hand with this study design we were able to include patients within a longer time period and there was sufficient clinical and follow-up data at hand because the patients were seen again in our outpatient clinic after discharge from angiography. Another limitation is the self-assessment of complications with the neuroradiologist revisiting the patient before discharge and the neuroradiologist seeing the patient again in the outpatient department after discharge.

As we retrospectively evaluated MRI studies that were performed as part of the clinical routine, the median time of MRI was 9 days after angiography. This might underestimate the true rate of DWI lesions.

The retrospective nature of the study also leads to a possible selection bias. There might have been high-risk patients that were intentionally angiographied in an in-house setting to avoid complications; however, this study presents the largest cohort of ambulatory patients undergoing cerebral diagnostic angiography.

Data collection and analysis to perform this study were approved by the local ethics committee. The study was performed in accordance with the ethical standards laid down in the 1964 Declaration of Helsinki and its later amendments.

## Conclusion

Complication rates for ambulatory diagnostic cerebral angiographies do not exceed those expected when angiography is performed with an overnight stay in hospital. Neither DWI lesions nor puncture site complications occurred at a higher rate. Our study showed no evidence of increased risks attributable to the ambulatory nature of the angiography. There was no symptomatic ischemic event after ambulatory diagnostic angiography. Thus, diagnostic cerebral catheter angiography can be performed safely in an ambulatory setting.
